# Electronic Tuning of Zinc Oxide by Direct Fabrication of Chromium (Cr) incorporated photoanodes for Visible-light driven Water Splitting Applications

**DOI:** 10.1038/s41598-020-66589-3

**Published:** 2020-06-16

**Authors:** Humaira Rashid Khan, Bilal Akram, Muhammad Aamir, Muhammad Azad Malik, Asif Ali Tahir, Muhammad Aziz Choudhary, Javeed Akhtar

**Affiliations:** 1 Materials Laboratory, Department of Chemistry, Mirpur University of Science and Technology (MUST), Mirpur, 10250 (AJK) Pakistan; 2 School of Materials, The University of Manchester, Oxford Road, Manchester, M13 9PL UK; 3 Environment and Sustainability Institute (ESI), University of Exeter Penryn, Cornwall, TR10 9FE UK; 4 Department of Chemistry, Tsinghua University, Beijing, 100084 China

**Keywords:** Chemistry, Inorganic chemistry, Composites

## Abstract

Herein, we report the synthesis of Cr incorporated ZnO sheets arrays microstructures and construction of photoelectrode through a direct aerosol assisted chemical vapour deposition (AACVD) method. The as-prepared Cr incorporated ZnO microstructures were characterized by transmission electron microscopy, scanning electron microscopy, energy dispersive X-ray spectroscopy, powdered X-ray spectroscopy, X-ray photoelectron spectroscopy and UV-Vis diffused reflectance spectroscopy. The Cr incorporation in ZnO red shifted the optical band gap of as-prepared photoanodes. The 15% Cr incorporation in ZnO has shown enhanced PEC performance. The AACVD method provides an efficient *in situ* incorporation approach for the manipulation of morphological aspects, phase purity, and band structure of photoelectrodes for an enhanced PEC performance.

## Introduction

Solar energy utilization has become the topic of research interest worldwide in order to cope with environmental issues and energy crisis^1,2^. Hydrogen generation as a clean fuel from solar energy through photoelectrochemical (PEC) water splitting is regarded as one of the most efficient strategy^3–5^. Since, Fujishima and Honda has demonstrated the use of TiO_2_ for PEC water splitting^6^, a variety of transition metals based semiconductors, such as metal oxides^7–13^, metal nitrides, and sulfides/selenides^14–16^, have been extensively investigated as photoanodes for PEC cells.

Among various semiconductors, ZnO has been widely explored as photoanodes due to its non-toxic, abundant, thermal and chemical stability and suitable optical band edges^17–23^. But, wide optical band gap of plain ZnO limits its large scale practical utility as it is only active in ultraviolet (UV) region of light. To widen its absorption window from ultra violet (UV) to visible region, among various strategies, the incorporation of other metal into plain ZnO thin films to modulate band gap is an effected strategy. ZnO thin films manifest interesting electrical and optical features upon incorporation of metal atoms. Various transition metals like Co^24^, Fe^25^, Mn^26^, Cu^27^ and Ga^28^ have been extensively investigated in terms of their incorporation into ZnO material. Among these, Cr has attracted significant attention^29–31^ because the incorporated Cr ions can significantly improve the optical characteristics of ZnO thin films by narrowing band gap energy. Meanwhile, the ionic radius of Cr^3+^ is quite close to the ionic radius of Zn^2+^, which reveals that Cr^3+^ can eagerly enter into the lattice and can replace Zn^2+^ in ZnO lattice^32^. The incorporated Cr atoms behave like donor species and provide free electrons. Conversely, this increases the concentration of free carriers, hence decreasing the electrical resistivity of the material.

Another way to improve PEC water-splitting performance by reducing electron recombination is to design new complex structural architecture of photoelectrode. Researchers are trying their best to explore new photoelectrode architectures^33^. Complex arrays such as 3D architectures are found to exhibit far enhanced PEC performance than other simple architectures^34^. However, the design of such architectures often requires sophisticated multistep processes. Therefore a direct fabrication of such photoelectrode architecture is highly desirable but still challenging^33^.

Herein, we report the direct fabrication of Cr incorporated ZnO 3D flower like structures assembled from nanosheets *via* a simple AACVD technique. The *in situ* incorporation of Cr has led to dramatic shape evolution as well as band gap modulation. The as-fabricated Cr incorporated ZnO thin films were characterized by powdered X-ray diffraction spectroscopy (pXRD), field emission scanning electron microscopy (FESEM), energy-dispersive X-ray spectroscopy (EDX), transmission electron microscopy (TEM), X-ray photoelectron spectroscopy (XPS) and diffused reflectance spectroscopy. Furthermore, the obtained photoanodes manifest significantly enhanced performance in PEC water splitting.

## Chemicals and Procedures

The chemicals and materials were purchased from Sigma Aldrich and used as received. The AACVD was used to fabricate plain and Cr^3+^ incorporated ZnO thin films on a glass substrate according to our previous reports^35–37^. In brief, different contents (2%, 5%, 10% and 15%) of Cr_3_O_4_ are mixed with Zn(CH_3_COO)_2_.2H_2_O in methanol (20 mL) to prepare the precursor solutions. The aerosols of resultant solution were generated by an ultrasonic humidifier. The aerosols were then transferred to the reaction chamber with the help of argon as a carried gas at 400 °C for 2 hours. Same protocol was adopted to perform deposition on FTO coated glass substrates. The resulting thin films were used to study the photoelectrochemical water splitting application.

## Characterizations

The as-fabricated thin films were characterized by different techniques as discussed earlier in the literature^36–38^. The morphological evolution of the as-fabricated thin films was explored by field emission scanning electron microscope (FESEM) (TESCAN MIRA3XMU, JEOL, USA) having an energy dispersive X-ray spectroscopy (EDX) instrument for elemental analysis. X-ray diffractometer (D8 ADVANCE XRD (Bruker, Germany) using Cu.K*α* radiation was used to determine the phase purity and crystallinity the as-fabricated thin films. The microstructures of as-fabricated thin films were confirmed by using Transmission Electron Microscopy (TEM) using Ion Company (FEI) Tecnai G2 F20 S-Twin microscope. X-ray photoelectron spectroscopy (XPS) was performed for the confirmation of the presence of Zn, O, Cr and to determine the oxidation state of Cr in incorporated ZnO material. The diffused reflectance of as fabricated thin films of plain and Cr incorporated ZnO (2%, 5%, 10% and 15%) was carried out by using SHIMADZU UV 1800 Spectrophotometer.

## Photoelectrochemical Set up

The photoelectrochemical water splitting studies were performed by using three-electrode system as described in our previous reports^37,38^. We used same procedure in present work. In this procedure, the counter, reference and working electrodes used were a Pt wire, Ag/AgCl and Cr-ZnO/FTO respectively, and 0.1 M Na_2_SO_4_ was used an electrolyte_._ The working electrode was dipped in the electrolyte solution to develop an electrical contact, whereas the undoped area was kept out of the electrolyte level with a gold-plate clip. The whole set up was exposed to light where it travelled through the electrolyte before reaching to the irradiated surface (~ 1.6 cm^2^) of working photoelectrode. The light source used in this particular experiment was AM 1.5 class A solar simulator (Solar Light 16 S - 300 solar simulator). A constant potential scan speed of 0.05 V/s used in all measurements. The PEC efficiency of as-fabricated photoelectrodes was performed by chopping light manually at regular intervals of 3 seconds/cycle^39^. A scan rate of 0.05 V/s in voltage range of 0.1 to 0.5 V was used to measure cyclic voltammetry (CV) curves. The chronoamperometric measurements was performed by chopping light irradiations for 900 seconds to determine photo stability and durability of photoanodes. All the electrochemical measurements were taken by using an Auto lab PGSTAT12 potentiostate.

## Results and Discussion

**Figure 1:**
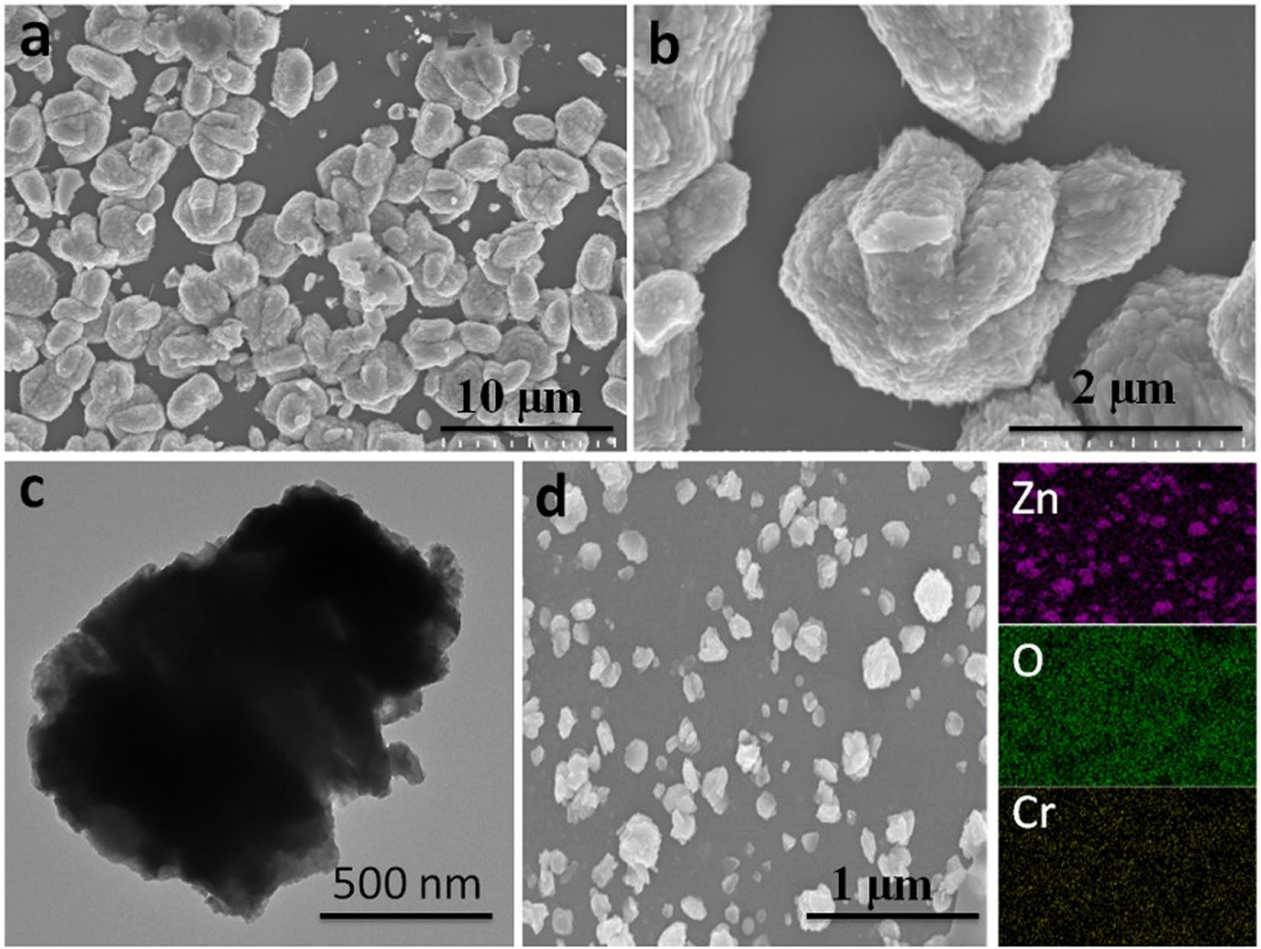
(**a**,**b**) FESEM images at different magnifications, whereas, (**c**) shows the TEM image and (**d**) is the elemental mapping through EDX of as-fabricated 15% Cr incorporated ZnO thin films.

The morphology of as-fabricated thin film microstructures was explored through FESEM and TEM. Figure 1a,b shows the FESEM micrographs of 15% Cr incorporated ZnO thin films microstructures. It can be seen that they are composed of numerous sheets like components grouped together in 3D flower like intricate patterns. TEM image in Fig. 1c also reveals a complex structure. The nature of the composite structure can be confirmed from the edges of this TEM image. It is interesting to note that all as-fabricated thin films have shown consistent and homogeneous microstructures. The elemental composition of the film microstructures was determined through EDX mapping (Fig. 1d) and it can be observed that all the deposited components (Cr, Zn & O) are evenly dispersed in the entire microstructures of the as-fabricated film.

**Figure 2:**
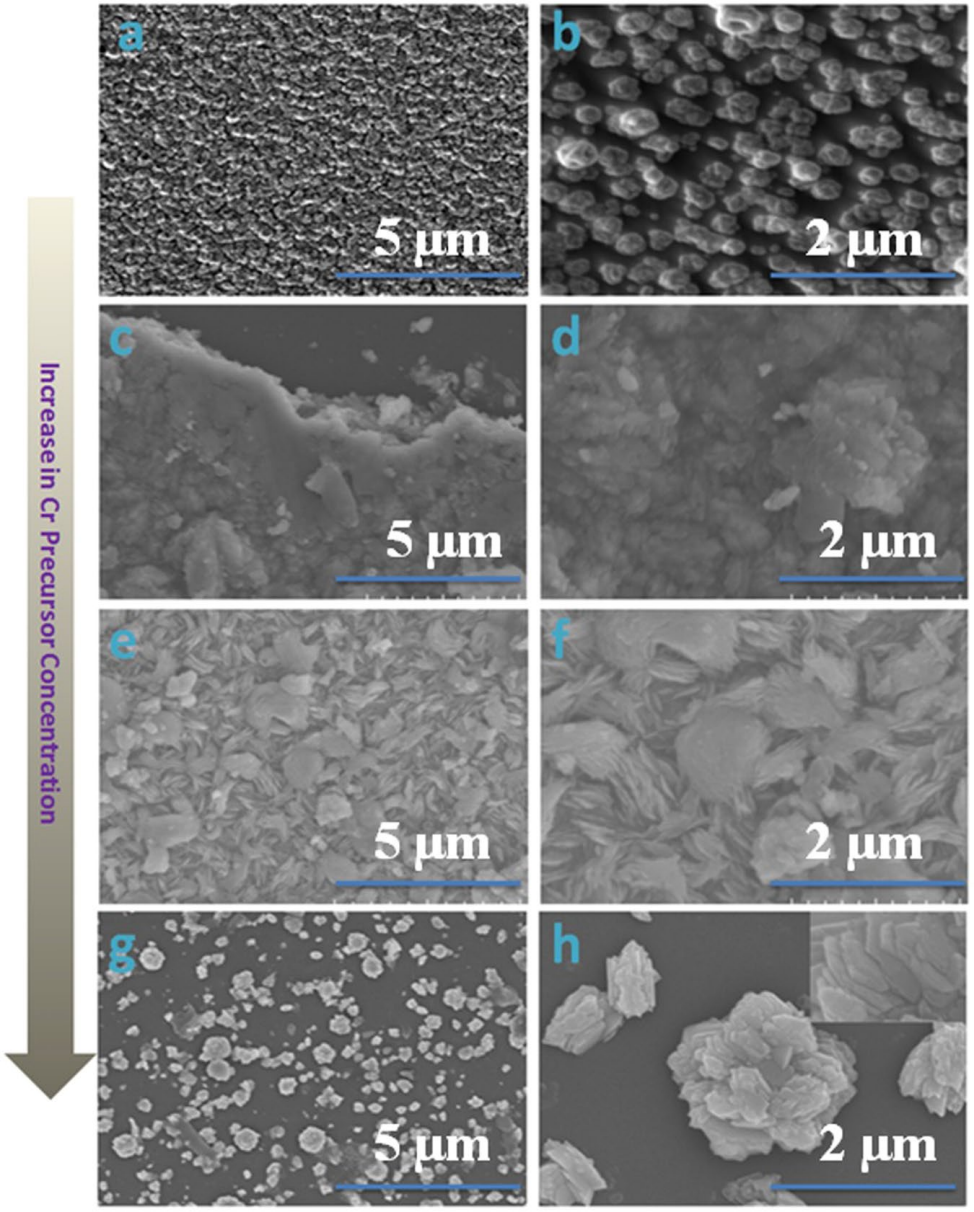
FESEM images of as-fabricated (**a**,**b**) plain ZnO, (**c**,**d**) 2% Cr incorporated ZnO, (**e**,**f**) 5% Cr incorporated ZnO, and (**g**,**h**) 10% Cr doped ZnO thin films.

Various controlled experiments with different molar ratios of chromium precursor were conducted to perform systematic study to explore the influence of Cr incorporation on the resulting microstructures of the films. Figure 2 reveals the SEM images of different films obtained with and without Cr incorporation. A uniform beads like structures were observed in plain ZnO films (Fig. 2a,b), which varies largely upon Cr incorporation. In case of 2% Cr incorporation, some irregular sheet like structures (Fig. 2c,d) can be seen which changes into regular stacked sheets (Fig. 2e,f) upon further increase in Cr concentration to 5%. As the concentration of Cr further increases to 10%, the complex flower like beautiful microstructure formed by the arrangements of stacked sheets (Fig. 2g,h). Based on the above observation, it can be inferred that the morphology of the nano crystallites changes from sheet like for 2% to complex flower like microstructures through intermediate stacked sheets. The morphological change depends on the fact that incorporation of Cr^3+^ into ZnO matrix causes variations in the growth through initial seedling. But still the exact mechanism for these variations is an open question.

According to Erwin model, ZnO thin films growing method is well understood by the oriented attachment mechanism. The (0001) plane of plain ZnO is positively charged. During this process, this plane of one growth unit combines with the negatively charged O-(0001) plane of another growth unit. The surface charges are compensated. The growth mechanism can be stopped at any time and consequently compact thin films of ZnO are deposited^40^. When Cr^3+^ is incorporated in ZnO lattice, it will replace the Zn^2+^ ions and the polarity of positively charged plane is increased. As a result, the combining ability of negatively charged plane of O with Zn-(0001) plane between different growing units is comparatively increased. Therefore, the ability of oriented growth is increased leading to the fabrication of the Cr-incorporated ZnO thin films^41^. Depending on the experimental results and reported literatures, it was observed that incorporation of Cr in ZnO lattice can create confined (localized) Cr energy states within the bandgap of ZnO due to which the energy band of ZnO is modified. When the as-fabricated photoelectrode is exposed to visible light, the photogenerated electrons from the valence band of ZnO can be transferred to the localized Cr energy states along with d-d transitions between the Cr incorporated energy states^42^. The photogenerated electrons can be readily trapped in the Cr dopant sites whereas the holes are left in the valence band of ZnO. Thus, by incorporating different concentrations of Cr as effective trapping sites in ZnO lattice, the photogenerated electrons and holes can be easily separated with limited chances of recombination of these photogenerated charge carriers, leading to the improved PEC performance. Briefly, we can say that when Cr metal is incorporated in the ZnO matrix, the electrons are accumulated at the interface between the metal and ZnO resulting in the downward band bending of the host side. This will allow the easy electron transfer from the surface of Cr to the ZnO side leading to enhanced PEC performance of Cr incorporated ZnO compared to plain ZnO photoanodes^43^.

**Figure 3:**
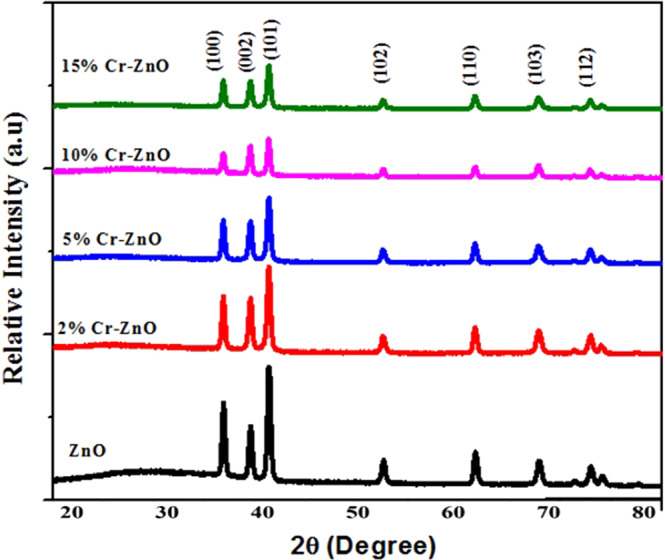
Comparative pXRD patterns of plain ZnO and (2%, 5%, 10% and 15%) Cr incorporated ZnO thin films.

The pXRD patterns of as-fabricated plain and Cr incorporated ZnO thin films are shown in Fig. 3. The diffraction peaks at 31.82°, 34.33°, 36.49° and 47.56° correspond to (100), (002), (101) and (102) planes of hexagonal wurtzite ZnO (ICDD: 086254). The peak intensities of plain ZnO observed in the Fig. 3 are high with (101) plane having maximum sharpness. As we move from plain ZnO thin films pXRD pattern to the Cr incorporated ZnO with different concentrations, the intensities of peaks decrease gradually. It can be observed that as the Cr content increases from 2% to 15%, the peaks for the incorporated ZnO thin films become weaker and wider, showing the inhibition of ZnO crystal growth by incorporated Cr. This trend suggests the formation of defects in the parent host matrix of ZnO crystal structure by the addition of Cr^44^.

**Figure 4:**
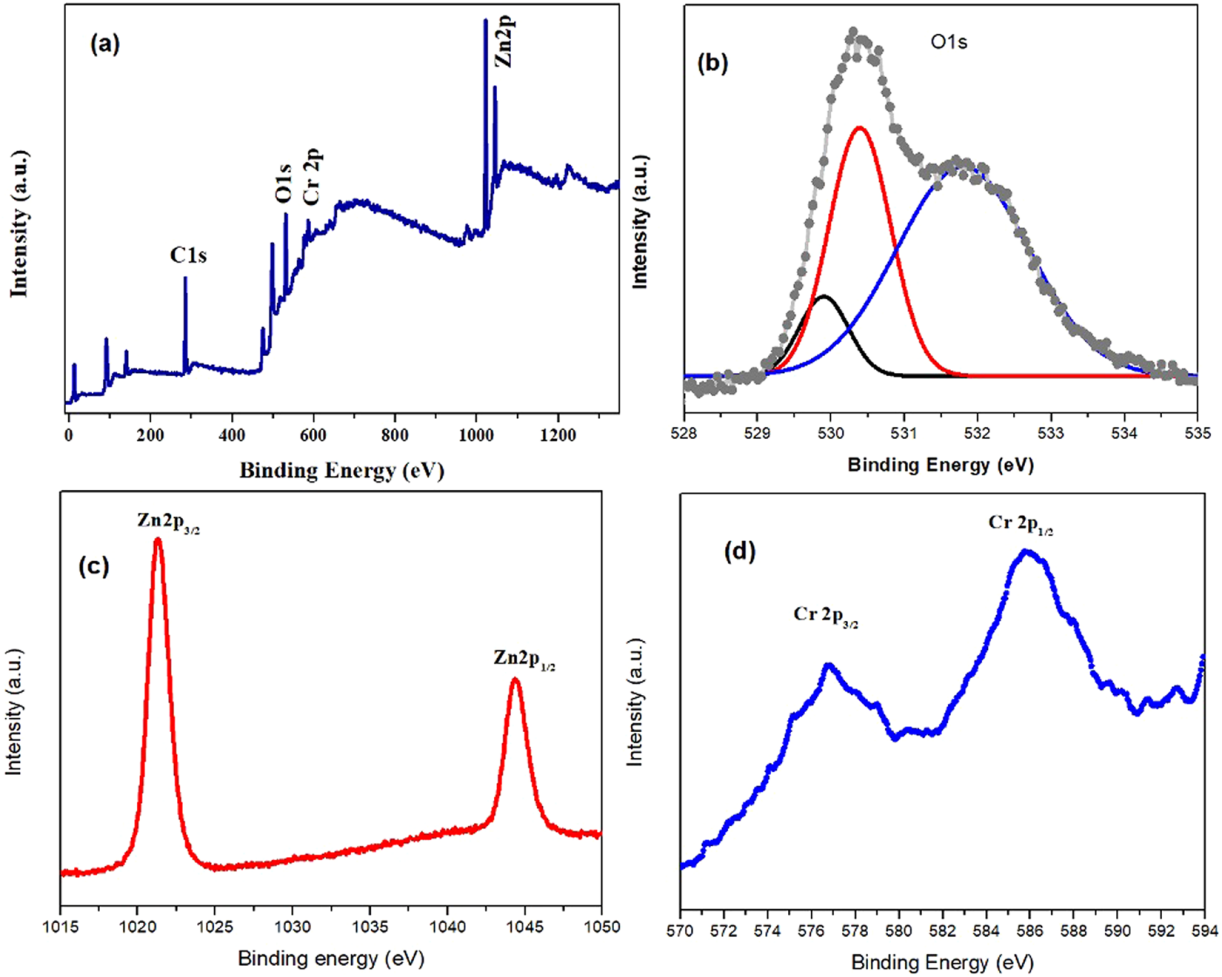
(**a**) XPS survey spectrum of Cr incorporated ZnO, while, (**b**) XPS high resolution spectrum of O 1 s, (**c**) Zn 2p, and (**d**) Cr 2p core levels showing the corresponding binding energy of O, Zn^2+^ and Cr^3+^ states.

X-ray photoelectron spectroscopy (XPS) was performed to confirm the presence of Zn, O, Cr and to ascertain the oxidation state of Cr in incorporated ZnO material. Figure 4(a) represents the survey spectrum of 15% Cr^3+^ incorporated ZnO films. The sharp peaks in survey spectrum belongs to O1s, Zn2p and Cr2p. The O1s contains three peaks located at binding energies of 529.8 eV, 530.4 eV and 531.8 eV as shown Fig. 4b. The peak of O1s at 529.8 eV represents the O^2−^ ions that assigned to crystal lattice of oxygen species with Zn ion enriched environment. Whereas, binding energy at 530.4 eV presents the surface adsorbed oxygen species like O^2−^ or O^1−^ or OH group of surface and oxygen vacancies. The binding energy of the 531.8 eV was allocated to the loosely bonded O ions on the surface of thin films like adsorption of H_2_O and O_2_^45,46^.

Figure 4c shows the peaks of Zn 2p_1/2_ and 2p_3/2_ states at 1044.35 eV and 1021.3 eV respectively, which were smaller than reported value of bulk value (1022.2 eV and 1045 eV)^47^ indicated the oxygen deficient 15% Cr^3+^ incorporated ZnO film^48^. The smaller values of binding energy in ZnO are due to Cr incorporation^45^. The Cr incorporated ZnO films have shown binding energy values at 585.85 eV for 2p_1/2_ and 576.75 eV for 2p_3/2_ as shown in Fig. 4d. These values for binding energy shows a marked difference from 574.2 eV for Cr metal and 576.0 eV for Cr^+2^, but it is close to the peak position of Cr 2p_3/2_ (576.7 eV) in Cr_2_O_3_^49^. It confirms the successful incorporation of Cr^3+^ in the ZnO matrix in the form of Cr^+3^ ions^50^.

**Figure 5:**
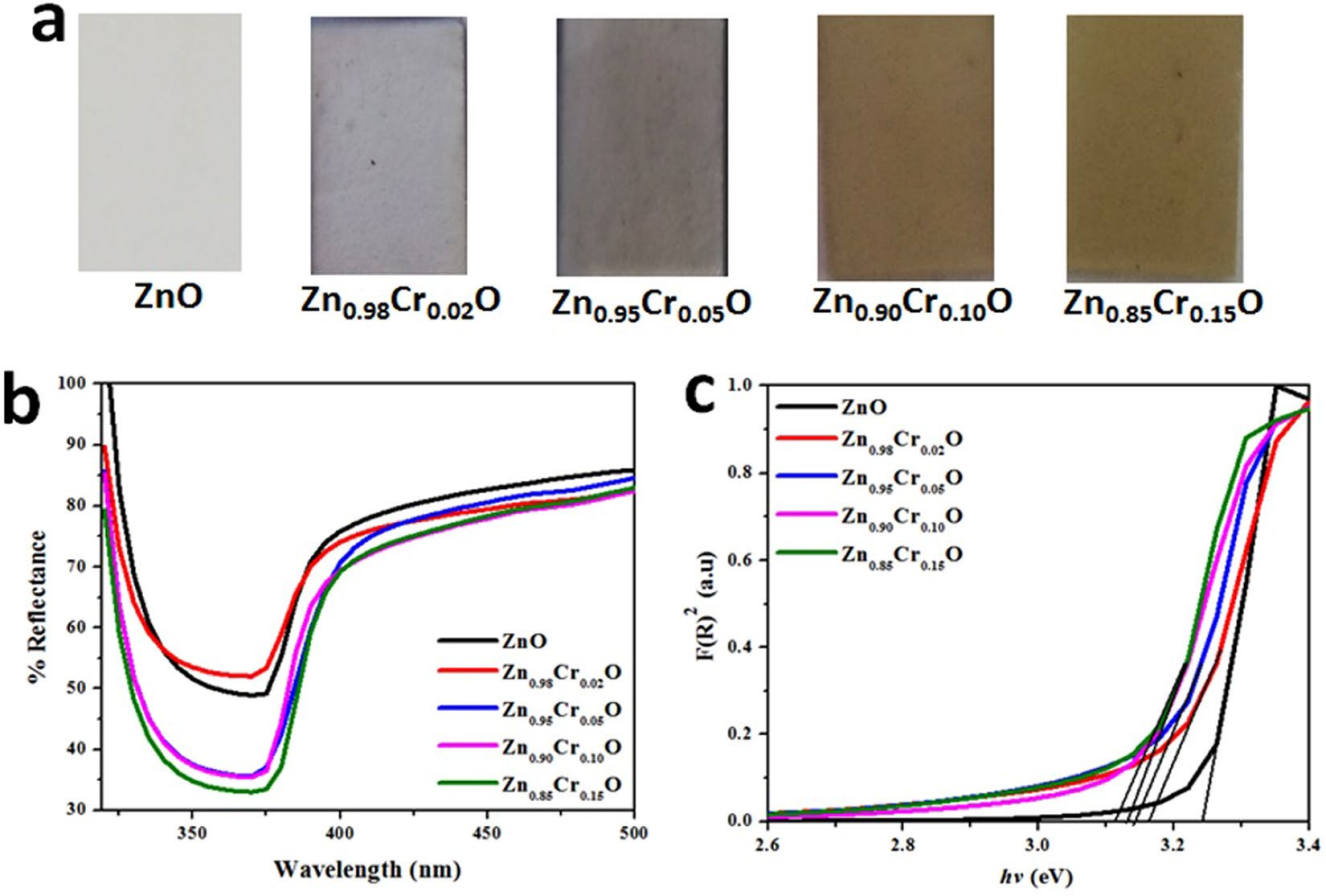
(**a**) Digital photographs of as-fabricated photoanodes, (**b**) diffused reflectance spectra of all as-fabricated thin films and (**c**) is the Kubelka-Munk plot for plain ZnO and Cr incorporated ZnO thin films.

Figure 5a shows the digital photograph of as-fabricated plain and Cr incorporated ZnO thin films. It can be seen that upon Cr incorporation the color of the films gets darker, which becomes deeper upon increase in Cr concentration.

The UV-Vis diffused reflectance spectra of plain ZnO and Cr incorporated ZnO thin films are presented in Fig. 5b. The light absorption properties of as-fabricated thin films changes with the incorporation of Cr in ZnO. As shown in Fig. 4b, the absorption edge of ZnO appears at 391 nm that shifted towards higher wavelength with increasing Cr content i.e. 400 nm for 15% Cr incorporated ZnO, resulting in the decrease in band gap of as-fabricated thin films. This may lead to increase in rate of electron-hole pair formation on the surface of substrate resulting in improved catalytic activity^51^. The energy band gap of as-fabricated thin films was estimated from diffused reflectance spectra using Kebula Munk method. For band gap calculations, the Kebula Munk factor, F(R)^2^ was plotted as the function of energy. It was found that band gap of as-fabricated ZnO thin films decreases with increasing the incorporated Cr content from 2% to 15%. Specifically, the energy band gap of as-fabricated thin films of pure and Cr incorporated ZnO (2%, 5%, 10% and 15%) were 3.24, 3.18, 3.14, 3.11 and 3.09 respectively as shown in Fig. 4c. This decrease in band gap is related to the s-d and p interactions of as-fabricated thin films^52^.

**Figure 6:**
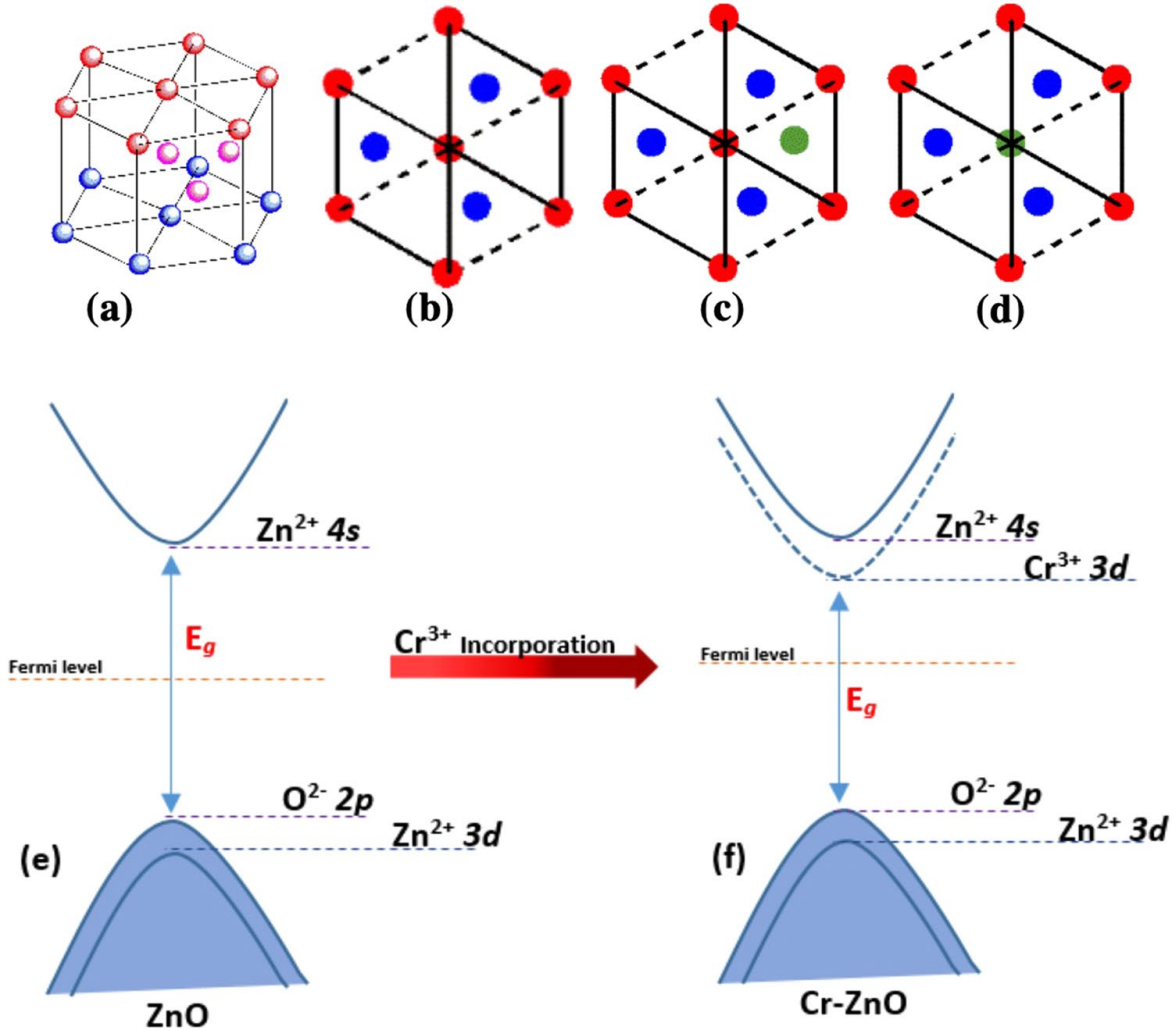
(**a**) Representative ZnO Wurtzite structure. The top view of (**b**) pure ZnO, (**c**) ZnO structure with substitutional incorporation with Cr^3+^, and (**d**) interstitial Cr^3+^ doping. (**e**) The band gap diagram of pure ZnO and (**f**) is the band gap diagram after Cr^3+^ incorporation into the ZnO structure.

The incorporation of Cr^3+^ ions into the ZnO wurtzite structure (Fig. 6a,b) can results in the placement of Cr^3+^ ions in two positions. The Cr^3+^ ions can occupy the interstitial sites that are octahedral coordinated sites and act as defects (Fig. 6c), however, more ideally, they substitute the Zn^2+^ ions and provide more charge carriers (Fig. 6d) and ultimate can increase the electronic properties of the ZnO material.

The Cr^3+^ incorporation in the ZnO host material have decreased the band gap of resultant compositions. As shown in Fig. 6e, the upper valence band (VB) of ZnO is made up of O 2p states and lower VB is made up of Zn 3d states. Whereas, the lowest conduction band (CB) is from the Zn 4 s states^53^. However, the Cr^3+^ incorporation in ZnO has shifted the fermi level upward into the CB (Fig. 5f), indicating the resultant materials (Cr-ZnO) is n-type^54^. Moreover, Cr^3+^ 3d states are further separated into two states, one lies across to the fermi level, while other lies above the fermi level. The electron intraband transition obtained due to the Cr^3+^ incorporation in Zn between VB and CB may leads to the intense absorption into the visible region^54^. These results have been supported by the optical absorption spectra as shown in Fig. 5. Similar results have also been reported by Kim et al^55^. for Fe-ZnO materials. On the other hand, the absorption coefficient has also been increased as shown in Fig. 4a, which is due to the Cr 3d impurity states. Based on these results, it can be proposed that the Cr incorporation in ZnO would increase the photoelectrochemical performance of ZnO due to the visible light absorption. Moreover, the absorption in UV region was also increased (Fig. 5a). Therefore, we have explored the photoelectrochemical performance of the as-fabricated Cr incorporated ZnO thin films.

## Photoelectrochemical Water Splitting

**Figure 7:**
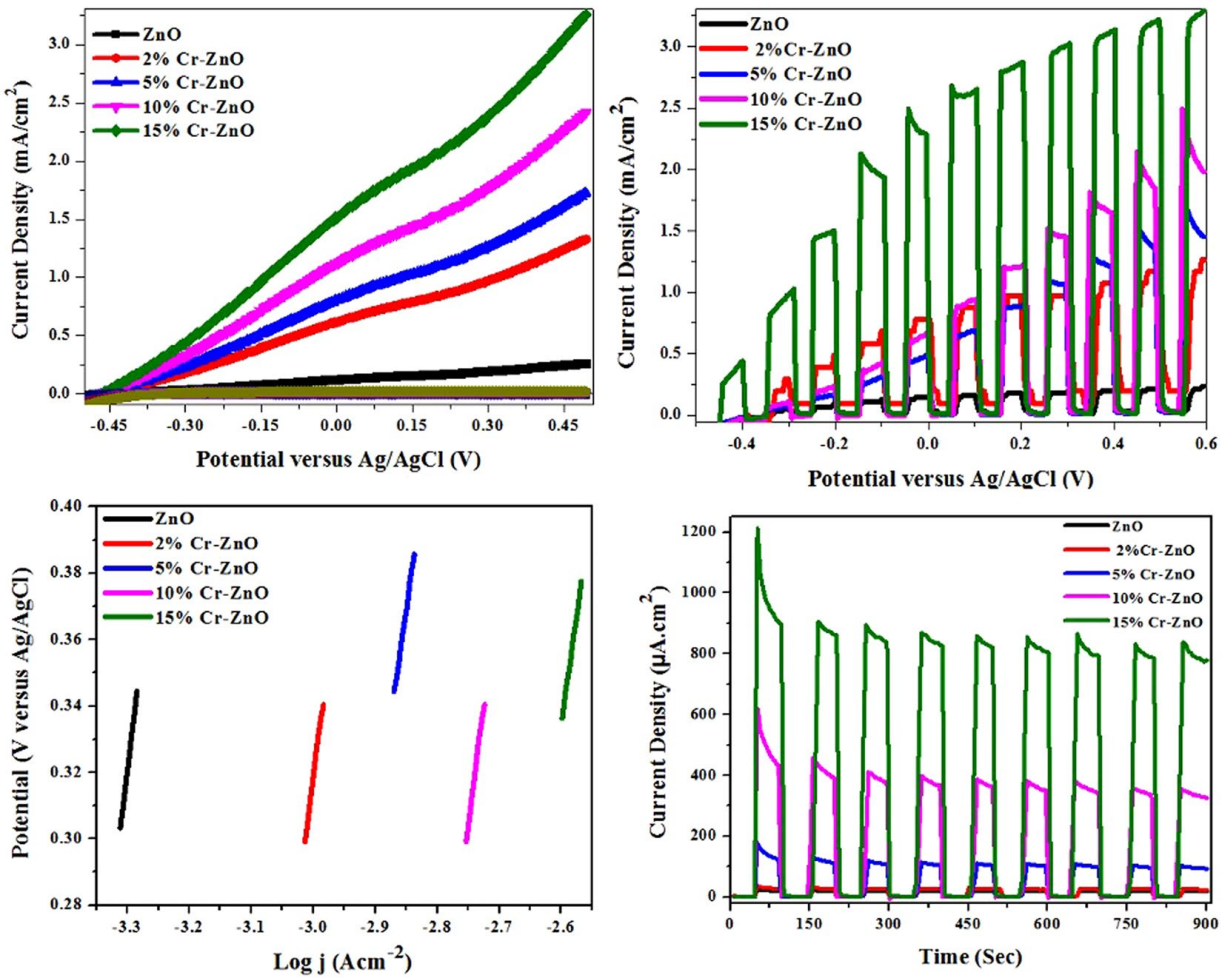
(**a**) Photocurrent potential curves of ZnO and Cr incorporated ZnO thin films under dark and light irradiation, where solid symbols reveals performance in light and hollow symbols shows photoelectrochemical behavior under dark, (**b**) current density under chopped light and dark conditions for plain ZnO and chromium incorporated ZnO photoelectrodes, (**c**) Tafel plots and (**d**) the chronoamperometric measurements for as-fabricated plain and Cr incorporated ZnO photoelectrodes.

The photoelectrochemical performances of as-fabricated thin films of pure and Cr incorporated ZnO were evaluated from the photocurrent-voltage (*I*-*V*) curves attained in 0.1 M Na_2_SO_4_ solutions both in dark and under UV irradiation and are shown in Fig. 7a. These linear sweep voltammograms were obtained from −0.5 V to +0.5 V versus Ag/AgCl. It was observed that in the presence of dark, all the photoelectrodes show negligible current. When photoelectrodes were exposed to the light irradiations, the photocurrent of plain and Cr incorporated ZnO photoelectrodes increased steeply at an onset potential of about −0.2 V. Upon irradiations, plain ZnO shows negligible current of about 0.25 mA/cm^2^. On incorporating different contents of Cr in ZnO lattice, the photocurrent increases gradually from 1.33, 1.76, 2.44 and 3.28 mA/cm^2^ for 2%, 5%, 10% and 15% respectively. This enhanced photocurrent values from plain ZnO to 15% Cr incorporated ZnO photoelectrodes confirm the presence of Cr as an efficient catalyst as compared to the plain ZnO photoelectrode.

The PEC activities of plain and Cr incorporated ZnO were further elaborated by the current-potential (I-V) curves under chopped irradiations as shown in Fig. 7b. The photocurrent density obtained for plain ZnO photoelectrode was 0.22 mA/cm^2^ that is quite negligible and can be attributed the absorption of visible light by the Zn^+2^ ions^56^. As compared to the plain ZnO, Cr incorporated ZnO photoelectrodes show a significant improvement in the photocurrent density from 1.28 mA/cm^2^ for 2% to 3.26 mA/cm^2^ 15% Cr incorporated ZnO photoelectrodes. This improved PEC activity for water splitting observed by the Cr incorporated ZnO photoelectrodes is essentially because of extended absorption of visible light by Cr incorporation into the ZnO lattice. The high photocurrent density of 15% Cr incorporated ZnO photoelectrode may be due to the complex morphology of as-fabricated thin films in addition to band gap effect and thus, gives best PEC activity.

The electronic properties and photoelectrochemical activity of ZnO photoelectrode is likely to be affected by the presence of Cr ion as an impurity. The increase in photocurrent density can be related to the reduction of Cr^+6^ to Cr^+3^ state in the visible-light absorption region of as-fabricated microstructure. It is clear from the previous literature that Cr^+3^ ions produce donor states in the band gap of ZnO that are related to the improved absorption of visible light that can help in enhanced photocatalytic evolution of hydrogen. On the other hand, Cr^+6^ ions decrease the photocatalytic performance due to their fast recombination centers^57,58^. In the present study, increased photocurrent can be observed after complete reduction of Cr^+6^ to Cr^+3^ in Cr incorporated ZnO microstructures that can be obviously confirmed from the Cr 2p XPS spectra.

To get more insights into the PEC activity for water splitting, the photoelectrochemical kinetics of as-fabricated photoelectrodes were evaluated by Tafel plots obtained from linear sweep voltammetry (LSV) curves. Figure 7c shows the Tafel plots for plain and Cr incorporated ZnO (2%, 5%, 10% and 15%) photoelectrodes. It can be observed that the Tafel slope values are 153, 148, 142, 131 and 125 mV/dec. for plain ZnO to 15% Cr incorporated ZnO photoelectrodes. The slope values decrease from plain ZnO to 15% Cr incorporated ZnO photoelectrodes which is important factor for the rapid catalytic activity of as-fabricated photoelectrodes^59^.

The chronoamperometric measurements were performed to check the stability of as-fabricated photoelectrodes in the presence of dark and light irradiation for 900 seconds as shown in Fig. 7d. When the light was turned on, the photocurrent observed for all the photoelectrodes increased to a maximum of about 28 µA/cm^2^ for plain ZnO to 866 µA/cm^2^ for 15% Cr incorporated ZnO photoelectrodes and reached a steady-state value. When the light was turned off, the photocurrent of all the as-fabricated photoelectrodes decreased and resumed to the initial value. The observed results depicted that the incorporation of Cr in ZnO not only increases the photocurrent density but also greatly improve the stability of the photoelectrodes.^60^ It is interesting to note that as the amount of Cr content increases from 2% to 15%, the photocurrent density as well as the stability of the photoelectrodes increases from 44 µA/cm^2^ for 2% to 866 µA/cm^2^ for 15% Cr incorporated ZnO. This improvement in current density is most likely due to the effective separation and transportation of charge carriers, which inhibits the photoelectrode corrosion on sudden illumination of light^61^. The stability observed by 15% Cr incorporated ZnO proved to be the best for PEC activity.

Thus, comparative photoelectrochemical performance of as-fabricated Cr^+3^ incorporated ZnO photoanode films on FTO by using AACVD method has showed that the 15% Cr incorporation in ZnO thin films has highest photocurrent and stability under light irradiation (Table 1). The high photoelectrochemical performance of the aforementioned photoanode was due to the band gap tuning of the ZnO material by expanding the absorption in the visible region, and also the enhancement in the absorption intensity.

**Table 1:** Comparative photoelectrochemical performance and other related parameters of as-fabricated photoanodes.

*Photoanodes*	Band gap (eV)	Photocurrent density (mA/cm^2^)	Stability (900 sec. irradiation) (µA/cm^2^)
*ZnO*	3.24	0.25	28
*2% Cr-ZnO*	3.18	1.33	44
*5% Cr-ZnO*	3.14	1.76	175
*10% Cr-ZnO*	3.11	2.44	400
*15% Cr-ZnO*	**3.09**	**3.28**	**866**

## Conclusion

In the present study, Cr incorporated ZnO thin film photoanodes have been fabricated *via* an effective and simple AACVD approach. *In situ* incorporation of Cr have proved to be a favourable factor for modulation of optical band gap energy, surface morphology and PEC water splitting performance of the resulting films. This work demonstrates the tailoring of electronic properties of ZnO thin films *via* controlled incorporation of Cr. This deposition strategy has provided the basis for the fabrication of variety of photoactive materials for potential light harvesting application. The customized composition and structure can be achieved simply by proper selection of precursor and manipulating their relative concentrations during growth. The as-fabricated photoanodes demonstrated moderate to good PEC activity and thus offering new dimension of research to explore the best composition of Cr doped ZnO photoanodes.
